# Exploration of Optimal Reaction Conditions for Constructing Hydrophobic Polymers with Low Deformation to Facilitate the Dimensional Stability of Laminated Bamboo Lumber

**DOI:** 10.3390/polym15122637

**Published:** 2023-06-09

**Authors:** Jianchao Zhou, Li Jin, Xinxing Wu, Hui Wang, Shuaibo Han, Yan Zhang, Fangli Sun

**Affiliations:** College of Chemistry and Materials Engineering, National Engineering & Technology Research Center for the Comprehensive Utilization of Wood-Based Resources, Zhejiang A&F University, Hangzhou 311300, China; 2020104041019@stu.zafu.edu.cn (J.Z.);

**Keywords:** laminated bamboo lumber, hydrophobic polymer, dimension stability, anti-swelling

## Abstract

The environmental moisture changes would result in the deformation and cracking of laminated bamboo lumber (LBL) easily due to the unreleased internal stress, leading to poor durability. In this study, a hydrophobic cross-linking polymer with low deformation was successfully fabricated and introduced in the LBL by polymerization and esterification to improve its dimensional stability. In an aqueous solution, the 2-hydroxyethyl methacrylate (HEMA) and Maleic anhydride (MAh) were employed as the base compounds for synthesizing the copolymer of 2-hydroxyethyl methacrylate and maleic acid (PHM). The hydrophobicity and swelling performance of the PHM was adjusted by controlling the reaction temperatures. PHM-modified LBL’s hydrophobicity as indicated by the contact angle, increased from 58.5° to 115.2°. The anti-swelling efficiency was also improved. Moreover, multiple characterizations were applied to clarify the structure of PHM and its bonding linkages in LBL. This study demonstrates an efficient avenue to facilitate the dimensional stability of LBL by PHM modification and sheds new light on the efficient utilization of LBL using a hydrophobic polymer with low deformation.

## 1. Introduction

Laminated bamboo lumber (LBL) has high strength, uniform structure, and flexible size characteristics, making it a popular material in furniture and construction. The green building material engineered bamboo has become increasingly important to modern construction due to its eco-friendliness and recyclable qualities [1,2]. LBL has been used to develop bamboo engineering products, compensating for bamboo’s inherent drawbacks [3]. However, a material derived from bamboo contains hydrophilic components and nutrients, which can cause cracking, deformation, and mildew when placed in alternately wet and dry environments [4,5]. These defects restrict bamboo’s development, which dramatically limits its outdoor application. Developing solid durable products would be LBL’s future focus [6]. However, LBL has not yet been extensively studied for durability, unlike raw bamboo and bamboo scrimber. Therefore, the damage caused by moisture changes to LBL must be prevented or limited [7].

Bamboo’s durability can be enhanced by increasing its hydrophobicity [8,9]. Bam-boo materials have been produced with hydrophobic or super-hydrophobic surfaces using poly-dimethylsiloxane (PDMS) lithography or micro-nano structure mimicking the lotus leaf structure [10]. However, internal modification is necessary for LBL outdoor applications due to the hydrophobic surface’s proneness to wear and weathering. Round bamboo was impregnated with polyethylene glycol and heated with paraffin, significantly improving its dimensional stability and hydrophobicity [11]. To improve bamboo’s dimensional stability, vinyl acetate, and methyl methacrylate were both studied for esterification and polymerization reactions, respectively [12]. Furfurylated bamboo obtained by vacuum-pressure impregnation displays high mold resistance [13]. A crosslinked copolymer network was constructed inside the wood to enhance its dimensional stability and reduce the possibility of cracks, the ASE (anti-swelling efficiency) of modified wood was in the range of 26.98% to 43.77% [14]. It has been found that impregnation methods and polymer modification can reduce the water-absorbing groups and fill cells effectively, which are considered effective methods to improve hydrophobicity and dimension stability [15,16].

The 2-hydroxyethyl methacrylate (HEMA) monomer has an unsaturated carbon-carbon bond and a hydroxyl group, which can be esterified to form crosslinking structures with other monomers and polymerization reactions, commonly used to modify resins and coatings [17,18,19]. The hydrophobicity of HEMA makes its homopolymers or copolymers suitable for modifying bamboo or wood. Bamboo was improved by in situ polymerization method in terms of dimensional stability and mold resistance, polyhydroxyethyl methylacrylate and polymethyl methylacrylate networks were used [20]. The poplar cell wall was in situ polymerized with HEMA and 3-(methacryloxy) propyltrimethoxysilane (MAPTES) to improve its dimensional stability [21]. A functional monomer of unsaturated double bonds and polar carboxylic groups, maleic anhydride (MAh) is commonly used in grafting and copolymerization [22,23]. Lignocellulosic materials can attain higher hydrophobicity and dimensional stability by removing hydroxyl groups from wood polymers with organic anhydride. MAh is a cyclic anhydride, and the esterification reaction of MAh generates carboxylic acid moieties on the wood and does not yield any undesirable by-products [24,25,26].

The copolymerization of HEMA and MAh can occur under certain conditions [27,28]. The material’s performance would be significantly enhanced by grafting a HEMA/MAh crosslinked network inside the LBL to the hydroxyl groups of bamboo by chemical bonds, with long-term effects. Anhydride and hydroxyl groups interact intensely in bamboo, which might facilitate polymer fixation [29]. HEMA and MAh can both be formulated into waterborne modifiers, which could facilitate bamboo penetration.

In this study, aqueous reaction systems using HEMA and MAh were used to prepare a crosslinked copolymer of 2-hydroxyethyl methacrylate and maleic acid (termed as PHM) in comparison to those using volatile organic solvents previously reported [27,28]. Ammonium persulphate (APS) was used as initiator and *N,N’*-Methylenebisacrylamide (MBA) as crosslinker. In an aqueous solution, MAh will hydrolyze to form maleic acid (MAc), which has a different reactivity. However, under dehydration conditions, MAc can be converted into MAh to obtain a higher reactivity [30]. Consequently, the reaction temperature was increased to promote the conversion of MAc to MAh and further improve the efficiency of the esterification reaction. The optimal PHM was consequentially built in situ in LBL for better hydrophobicity and dimensional stability. Furthermore, the PHM-modified LBL properties and internal bonding linkages were also evaluated to explain how PHM can enhance LBL properties.

## 2. Experimental

### 2.1. Materials

The LBL was obtained from Dasso bamboo technology, Ltd., Fujian, China, and sawed into samples with dimensions of 20 mm (longitudinal) × 20 mm (radial) × 5 mm (tangential) for the experiments.

MAh and APS were purchased from Aladdin Co., Ltd. (Shanghai, China). HEMA and MBA were provided by Macklin Co., Ltd. (Shanghai, China). All chemicals were analytical grade or higher and used without further purification.

### 2.2. Synthesis of PHM

HEMA and MAh monomers at a molar ratio of 3:2 were dissolved in deionized water to prepare a 40 wt. % monomer solution. 0.6 wt % APS and 0.4 wt % MBA of the total monomer amount were added into the solution, followed by sufficiently stirring until a homogeneous reaction solution was obtained. Subsequently, 30 g solution was weighed into each glass container and reacted at 100, 120, 140, 160, and 180 °C, respectively, for 2 h. The resulting polymers were correspondingly designated as PHM-100, PHM-120, PHM-140, PHM-160, and PHM-180.

### 2.3. Characterization

The PHMs for characterization were firstly extracted with a 2:1 (volume ratio) mixture of ethyl alcohol and deionized water to remove the unreacted monomers, then dried at 80 °C to constant weight before characterization. The extracted polymer samples were fractured in liquid nitrogen to obtain smooth sections. Then homogeneous gold layers were sputter-coated on their sections. The microstructure of PHM was observed using the TM3030 (HITACHI, Tokyo, Japan) scanning electron microscope (SEM) at a voltage of 1.5 kV.

Fourier transform infrared (FT-IR) spectra were recorded using the IR Prestige-21 Spectrophotometer (Shimadzu, Kyoto, Japan) in the range of 500-4000 cm^−1^ with a 4 cm^−1^ resolution and 32 scans. The copolymer samples were prepared as spectroscopic quality KBr pellets with a sample/KBr ratio of 1/100. ^13^C-NMR analysis was performed at 400 MHz (Bruker, Bremen, Germany) using solid powder samples. The glass transition temperatures (T_g_) of PHM were determined at differential scanning calorimetry (DSC) measured with a Q2000 thermal analyzer (TA Instruments, New Castle, DE, USA) under a nitrogen atmosphere. Each sample was heated from −70 °C to 150 °C at 10 °C/min.

### 2.4. Evaluating the Performance of PHM under Water Immersion

The percentage of water absorption (*P_w_*) and volume swelling (*P_v_*) of PHM were determined through three cycles of water immersion-oven dry (W-D) procedure. Samples were not extracted before the test. In each process, samples were soaked in deionized water at room temperature for 24 h, then dried at 80 °C to a constant weight. The mass and volume variation was recorded after soaking or drying. *P_w_* and *P_v_* were calculated from Equation (1) and Equation (2), respectively. Additionally, the average volumetric gain per unit water absorption (*VG*) based on three cycles test was computed to evaluate the dimensional stability of the polymer upon contacting water.

(1) $$P_{w}=\frac{M_{w}-{\;M}_{d}}{M_{d}}\;\times \;100\%$$

(2) $$P_{v}=\frac{V_{w}-{\;V}_{d}}{V_{d}}\;\times \;100\%$$

(3) $$VG\;=\frac{P_{v}}{P_{w}}$$

In the equations, *M_d_* and *V_d_* represent the mass and volume of the specimens after drying, and *M_w_* and *V_w_* are data after water absorption.

In addition, the average mass loss ratio (*ML*) of the copolymers was calculated based on the three cycles swelling experiment to show their leaching resistance when exposed to water immersion. The specific formula is as follows:

(4) $$ML=\frac{M_{1\;}-{\;M}_{2}}{M_{1}}\times 100\%$$

In the above equations, *M*_1_ and *M*_2_ represent the samples’ dry weight before and after soaking.

### 2.5. Construction of PHM in LBL and Its Dimensional Stability

The reaction solution (40 wt. %) prepared as described in Section 2.2 was impregnated into LBL blocks under a vacuum of 0.09 MPa for 15 min and pressurized at 0.8 MPa for 30 min. The impregnated LBL was then heated at designated temperatures (100, 120, 140, 160, 180 °C) for 2 h to complete the polymerization. Then, LBLs were extracted with a 2:1 (volume ratio) mixture of ethyl alcohol and deionized water to remove the unreacted monomers and dried at 80 °C to constant weight before tests.

The contact angle characterizes the hydrophobicity of modified LBL conducted using a video optical contact angle tester (*OCA50AF, Dataphysics, Filderstadt, Germany). The deionized water was dropped on the sample surface at 9 different sites. A fixed droplet volume of 5 μL deionized water was used, and the test duration was set at 3 s. The contact angle was measured and averaged to obtain the contact angle of LBL.

LBLs were soaked in water for 72 h to simulate the moisture circumstances, allowing full saturation at the moment of measurement in the soaked state. The dimensional stability is characterized by the swelling ratio (*S_w_*) and *ASE* after water absorption. 8 samples were used for the test.

(5) $$S_{w}=\frac{V_{w}-V_{d}}{V_{d}}\times 100\%$$

(6) $$ASE=\frac{S_{0}-S}{S_{0}}\times 100\%$$

*V_d_* and *V_w_* represent the volume of the test block before and after water absorption, respectively. *S* is the swelling ratio of modified LBL, and *S_0_* is the data of raw LBL.

## 3. Results and Discussion

### 3.1. The Water Absorption and Swelling Behaviors of PHM

**Figure 1:**
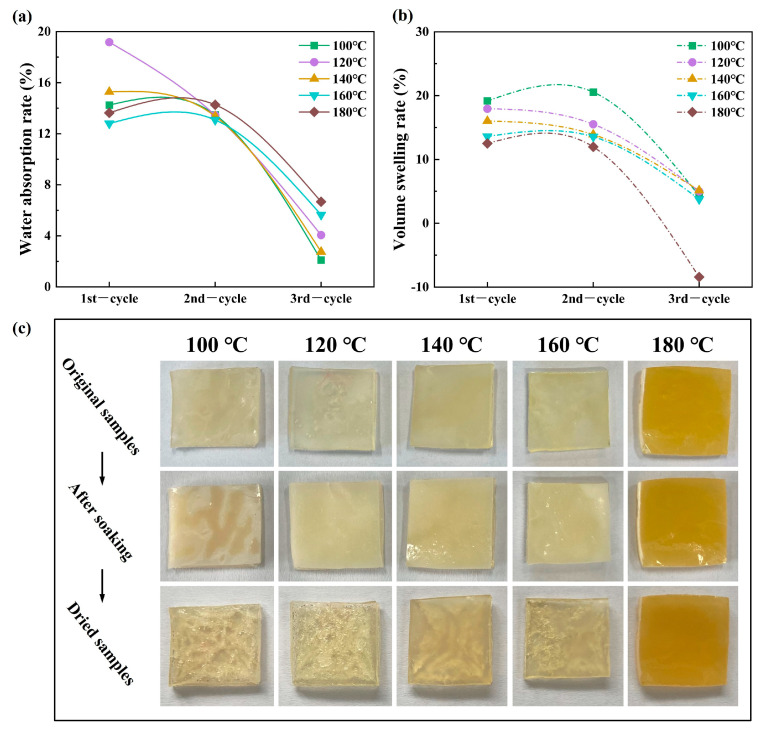
Percentage of water absorption (**a**), volume swelling rate (**b**), and changes in the appearance of PHM (**c**) during three cycles of the water immersion-oven dry procedure.

To obtain an optimal crosslinked polymer with excellent hydrophobic properties for LBL modification, the reaction temperature that initiated both the polymerization and esterification of HEMA and MAc was paid special attention. Figure 1 presents polymer samples’ water absorption and swelling behaviors during the three water immersion-oven dry (W-D) procedure cycles, reflecting the hydrophobicity and deformation resistance. Overall, polymers’ *P_w_* and *P_v_* decreased gradually in three cycles. During the first two cycles, PHM showed more than 12% water absorption capacity, causing a volume swelling rate exceeding 10%. The hydrophilic functional groups can generate strong interactions with the swelling medium to cause water absorption and volume expansion [31]. Conversely, the initially stable crosslinking network presented hydrophobicity after two cycles of water-dry procedures, with *P_w_* dropping significantly, in the range of ~2.1 to ~6.7%, and correspondingly, *P_v_* diminishing below 5.1%. The decreased water absorption and volume swelling might be ascribed to the leaching of unreacted monomers and low molecular non-crosslinked polymers. The formed three-dimensional crosslinking network could improve PHM’s cohesive strength and decrease the swelling rate. The effect of reaction temperature on the *P_w_* and *P_v_* was not as evident as expected, especially for the *P_v_*. Except for PHM-180, the *P_v_* remained almost the same in the 3rd cycle. No volume swelling occurred to PHM-180 on the third water immersion cycle, indicating a stable dimension for PHM synthesized at 180 °C.

Nevertheless, high-temperature products showed significant advantages in resistance against deformation, especially PHM-160 and PHM-180. The appearance of the samples can also indicate that the higher reaction temperature causes lower water absorption during soaking and reduced cracking after drying the samples (Figure 1c). PHM-180 samples exhibited a darker color due to a significant increase in the number of ester bonds and the possible oxidation reaction.

Table 1 also provides information about polymers’ volumetric gain per unit water absorption (VG) and mass loss ratio (ML) during the swelling test. The values of VG are around 1.0 except for PHM-100 and PHM-140, indicating a synchronized gain in water absorption and volume swelling, which is essential in stabilizing the dimension of LBL. Moreover, the overall trend of VG declined as the temperature increased, indicating that the volume expansion of polymers synthesized at lower temperatures was more pronounced when absorbing the same amount of water. The relatively loose structure of these polymers was more easily destroyed by water. Polymers with an excellent performance by the higher crosslinking density will not yield noticeable volume variation even applied to a water immersion or highly humid environment, which lays a foundation for modifying LBL. The low VG could be attributed to the stable and firm structure forming from crosslinking and esterification reactions, also probably caused by the mass loss-induced shrinking. The ML values displayed little relevance to the temperature, which might be attributed to the coexistence of polymerization and hydrolysis in the aqueous reaction system. However, no correspondence between VG and ML could be concluded. Comparatively, PHM synthesized at 160 °C is lower in both VG and ML than PHM obtained at different temperatures, very suitable for LBL modification. PHM-160’s excellent performances benefit from its hydrophobic crosslinking network.

As a water-soluble monomer, HEMA can homopolymerize into PHEMA and copolymerize with another monomer, such as MAc [32]. PHEMA is a hydrophilic polymer, with *P_w_* as high as 25% and *P_v_* 30% [33]. MAc can copolymerize with HEMA to form PHM. PHEMA and PHM can form a crosslinked network structure in the presence of MBA, and the plentiful hydroxyl groups and carboxyl groups can react with each other to produce hydrophobic ester bonds under high temperatures and the continuous removal of water. A higher reaction temperature contributed to the esterification efficiency, as water is apt to discharge [27,34]. Meanwhile, MAc would be converted into MAh (see Supplementary Materials), which promoted the esterification between HEMA and MAh, and further improved the crosslinking density. The results in Figure 1 and Table 1 presented an optimal reaction temperature at 160 °C for the fabrication of an overall dimension stable PHM, which will be applied in the modification of LBL.

### 3.2. Characterization of PHM

**Figure 2:**
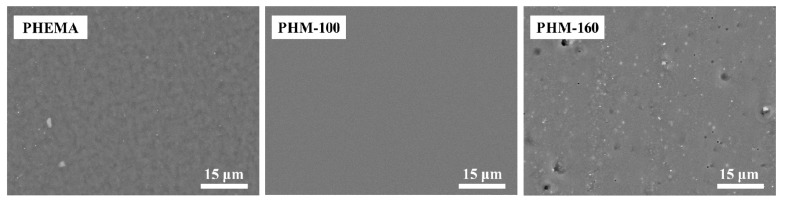
Fracture morphology of PHEMA and PHM.

The properties of the material are closely related to its composition and microstructure. Therefore, it is vital to understand the microstructure of obtained polymers and clarify the internal relationship between structure and properties. Figure 2 shows a homogeneous but rough morphology for the HEMA homopolymer (PHEMA) was observed. The wrinkled surface was probably related to its brittleness during the drying procedure [35]. In contrast, HEMA/MAc copolymer (PHM) showed a smooth and uniform appearance without apparent phase separation, which indicates good compatibility between HEMA and MAc before and after the reaction. As both monomers dissolved well in an aqueous solution, they would react to form the copolymer. Besides, esterification could occur between the hydroxyl group on HEMA and the carboxy group on MAc. Consequently, a crosslinked structure with better continuity and homogeneity was formed through esterification and copolymerization, providing enhanced toughness.

Compared with PHM reacted at 100 °C (PHM-100), a more rough and porous structure was observed on PHM at a reaction temperature of 160 °C (PHM-160), which could be ascribed to the violent reaction at high temperature, particularly in an aqueous system. Once the water evaporated from the reaction solution, the system became viscous. The copolymerization of HEMA and MAc further increased the system’s viscosity, MAc tended to convert to MAh. At that moment, a reaction temperature higher than 100 °C accelerated the esterification reaction, resulting in water vapor. A more porous structure would thus be formed. Moreover, the grey phase shown as tiny dots in PHM-160 might be the esterification of HEMA and MAh, which induced slight phase separation [36].

**Figure 3:**
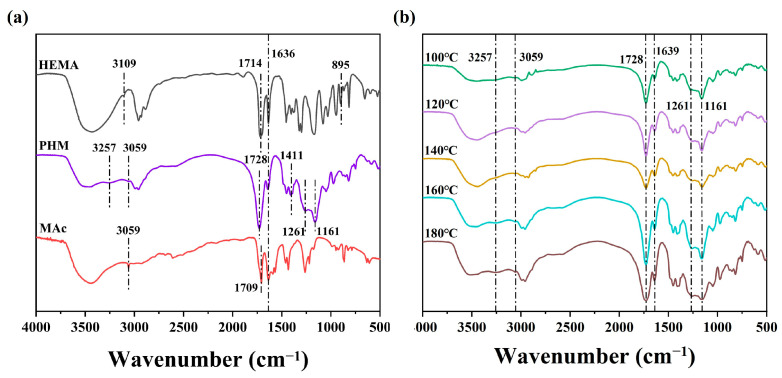
FT-IR spectra of HEMA, MAc and PHM (**a**); FT-IR spectra of PHMs synthesized at various temperatures (**b**).

In order to determine the chemical reaction between monomers HEMA and MAc, FT-IR analyses were performed on both monomers and the reaction product PHM shown in Figure 3a,b. The vinyl vibration peak appearing at 3059 cm^−1^ and 1636 cm^−1^ decreased in the spectrum of PHM due to polymerization [37]. The N–H stretching vibration peak at 3257 cm^−1^ and the C−N absorption peak at 1411 cm^−1^ appeared owing to the groups on the segment of MBA crosslinking sites [38,39].

The esterification of HEMA and MAc in PHM could also be detected from the specific FT-IR bands. The decreased band of hydroxyl groups around 3600–3200 cm^−1^ compared with the monomers probably resulted from the consumption of –OH during esterification [40]. At 1714 cm^−1^ and 1709 cm^−1^, a C=O stretching vibration is observed for HEMA and MAc, whereas 1728 cm^−1^ is for PHM. The significantly shifted peak of C=O also suggested the formation of an ester bond. Additionally, the absorbances at 1261 cm^−1^ and 1161 cm^−1^ characterize the ester C–O bond enhanced in PHM, further confirming the esterification reaction between HEMA and MAc [41,42]. These bands were found to be intensified when the reaction was conducted at a higher temperature, especially 160 °C and 180 °C (Figure 3b), which related to the discharge of water and the high reactivity of MAh. The increase in ester bonds in the polymer established that elevated temperature facilitated the esterification reaction.

**Figure 4:**
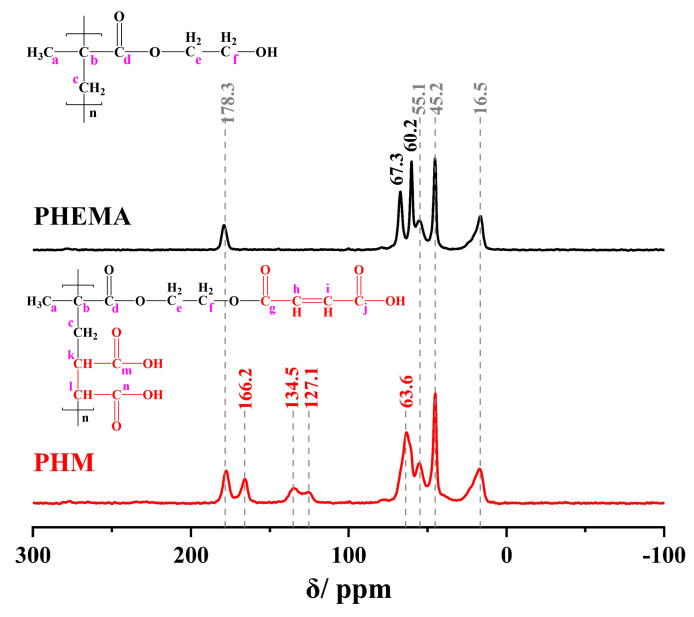
^13^C-NMR spectra of PHEMA and PHM synthesized at 100 °C.

The result of ^13^C-NMR became an essential corroboration of the characterization of the copolymer combined with the FT-IR analysis. The spectral features of PHEMA and copolymer PHM are presented in Figure 4.

On the spectrum of PHM, carbon signals on leading chains appeared at 55.1 ppm (c,k) and 45.2 ppm (b,l), and –CH_3_ carbon was located at 16.5 ppm. The signals at 178.3 ppm and 166.2 ppm were attributed to Carbonyl (C=O) of HEMA and MAc groups, respectively. There are both HEMA segments and MAc segments in PHM. In addition, absorption at 67.3 ppm and 60.2 ppm were attributed to –CH_2_ (e,f) of HEMA groups. Due to the esterification of the –OH group, this signal appeared at 63.6 ppm on the PHM spectrum. Unsaturated carbons of C=C in MAc were observed at 127.1 ppm and 134.5 ppm [43,44,45].

**Figure 5:**
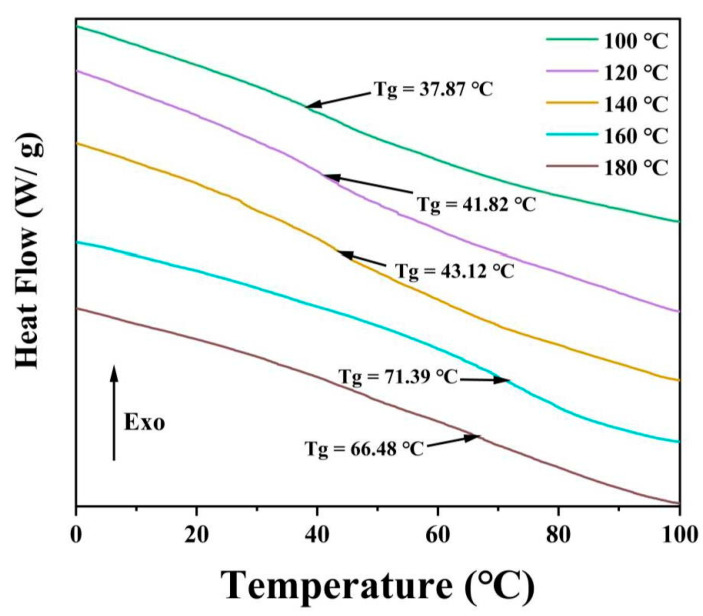
Differential scanning calorimetry (DSC) spectra of PHMs.

Glass transition temperatures based on differential scanning calorimetry measurements were used to investigate the effect of reaction temperature on the cross-linkage of PHM. As shown in Figure 5, T_g_ significantly increased with the reaction temperature raised to 160 °C (T_g_ = 71.39 °C). The increase in T_g_ was attributed to the restrained mobility of polymer chains [46], which resulted from the improved degree of esterification. The -COOH groups on MAc with the –OH groups on HEMA could form ester bonds under heating conditions, and the rise of temperature was beneficial to water discharge to generate more ester bonds. Furthermore, MAc can easily be converted to MAh above 140 °C (see Supplementary Materials), providing a higher reactivity. The number of free hydroxyl and carboxyl groups significantly reduced, leading to decreased fluidity in the molecular chains and reduced free volume in the copolymer network. A study of DSC analysis showed that the elevated temperature facilitates the cross-linkage of PHM in agreement with the above FT-IR analysis.

**Figure 6:**
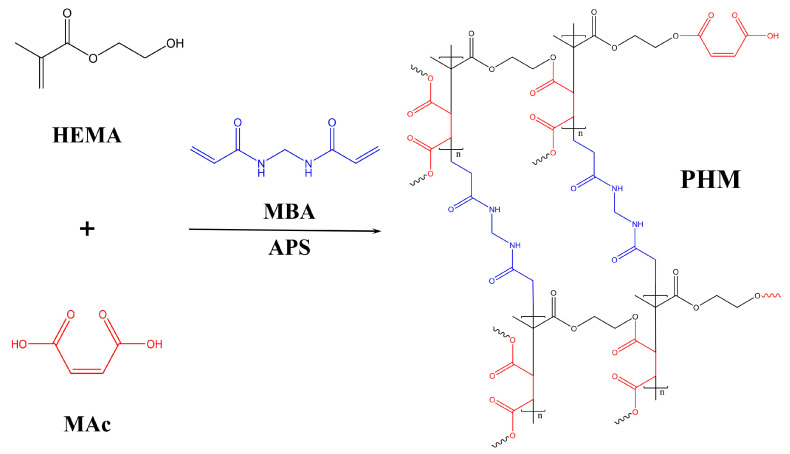
Schematic representation of crosslinking reactions between monomers.

Based on the comprehensive analysis above, the elevated temperature is more propitious to obtain a polymer with higher hydrophobicity and dimensional stability, and 160 °C is considered the optimized reaction temperature. The crosslinking reactions are shown in Figure 6.

### 3.3. Hydrophobicity and Dimensional Stability of the PHM-Modified LBL

**Figure 7:**
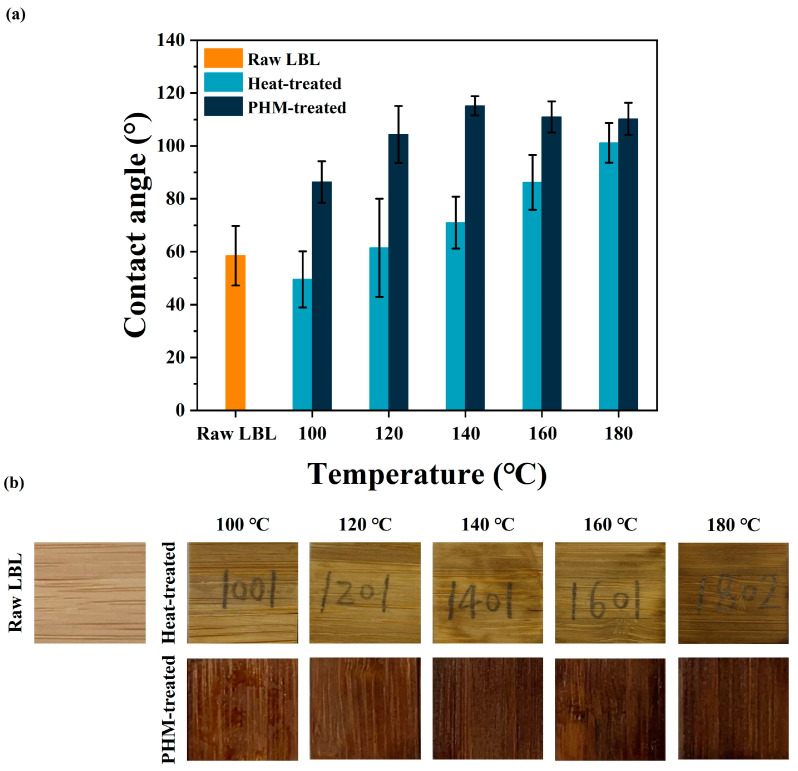
Water contact angles (**a**) and surface color of treated LBL concerning different conditions (**b**).

Based on the analyses of PHM synthesized at different temperatures, the reaction solution was immersed and in situ reacted in LBL. Hydrophobicity was assessed by measuring the water contact angle (WCA) of the modified LBL generated by polymerization, crosslinking, and esterification of HEMA and MAc (as shown in Figure 7). Since heat modification is also an effective method of reducing the hygroscopicity of bamboo [47,48], contact angles were measured for both heat-treated and PHM-treated blocks, with the former as the control. Due to numerous hydroxyl groups, the raw bamboo had a low water contact angle, which was 58.5°. However, since the number of hydrophilic groups reduced due to the thermal modification, the surface wettability significantly decreased, and the higher temperature resulted in higher contact angles (Figure 7a). The hemicellulose was hydrolyzed after the heat treatment, and the higher the temperature, the more intense the hydrolysis reaction. Degradation of the hemicellulose reduced available free polar adsorption sites, which was the main reason for the increasing hydrophobicity [49]. The heat-treated blocks at 100 °C showed a lower contact angle than untreated ones, this might be caused by the rising infiltration due to cracks on the bamboo surface. Compared to the heat-treated method, the hydrophobicity of PHM-treated materials was sharply enhanced. PHM had a crucial effect on the hydrophobicity modification of LBL. The WCAs achieved a peak value (115.2°) when the temperature reached 140 °C. When the temperature further increased, hydrophobicity changed inconspicuously. The reason might be that the hydroxyl esterification of bamboo by the synergistic effect of high temperature and polymer had reached saturation. Moreover, it can be discovered that the color of both heat-treated bamboo and PHM-treated ones became dark in comparison with the control, and the latter was more profound than the former due to the combination of heat treatment and resulted in polymers formed in bamboo, as well as esterification of MAc/MAh and hydroxyl groups in bamboo [46].

**Figure 8:**
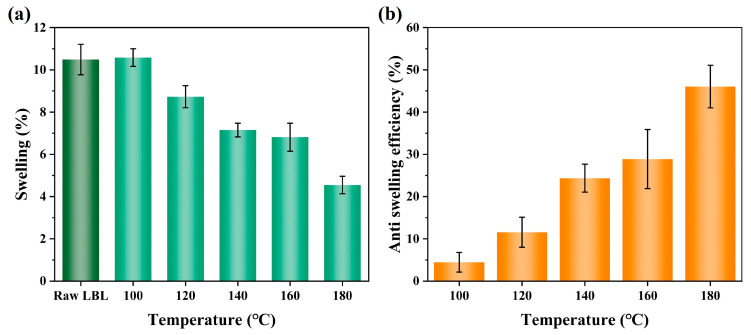
Swelling ratio (**a**) and anti-swelling efficiency (**b**) in the soaking experiment of LBL.

As a porous and hydrophilic material, water invasion does not exclusively occur on the bamboo’s surface. When LBL is applied to a humid environment, the hygroscopic components of the cell wall in the bamboo will absorb moisture to expand, resulting in changes in the size of the cell wall or even the entire material, especially in the transverse directions, leading to a series of problems. During the water immersion procedure, modified LBL resisted wet expansion, especially when the reaction temperature reached 140 °C. The untreated LBL exhibited a significant expansion due to its hydrophilicity, and *S_w_* is 10.5% (Figure 8). The high swelling ratio and low ASE of LBL treated under 100 °C were related to the poor crosslinking and the high VG of PHM. Furthermore, the esterification between maleic acid and bamboo was limited, the unreacted MAc had a negative effect on bamboo, resulting in increased hydrophilicity. Unlike surface hydrophobicity, LBL’s dimensional stability improved when the reaction temperature increased to more than 140 °C. The swelling ratio was as low as 4.5%, and ASE achieved 46.0% when the temperature reached 180 °C. The high temperature may lead to a reversion of MAc back to MAh state to permit a better level of cross-esterification between the MAh and the HEMA or the MAh and the bamboo. The improved dimensional stability reflected the importance of internal modification.

### 3.4. Mechanism of Action of PHM on LBL’s Hydrophobicity and Dimensional Stability

The reaction monomers were diffused into the interior of bamboo by vacuum-pressure impregnation. PHM could consume the free hydroxyl groups and increase its surface hydrophobicity when constructed in the material. When the monomer solution diffused into the bamboo cell wall, for one thing, MA (MAc or MAh) chemically bonded to the hydroxyl groups of bamboo components, reducing the number of free hydroxyl groups and weakening the diffusion of water molecules into the bamboo cell wall [27], many reports indicate that MAh has been used to modify lignocellulosic materials via esterification reactions and achieve good results [50].

**Figure 9:**
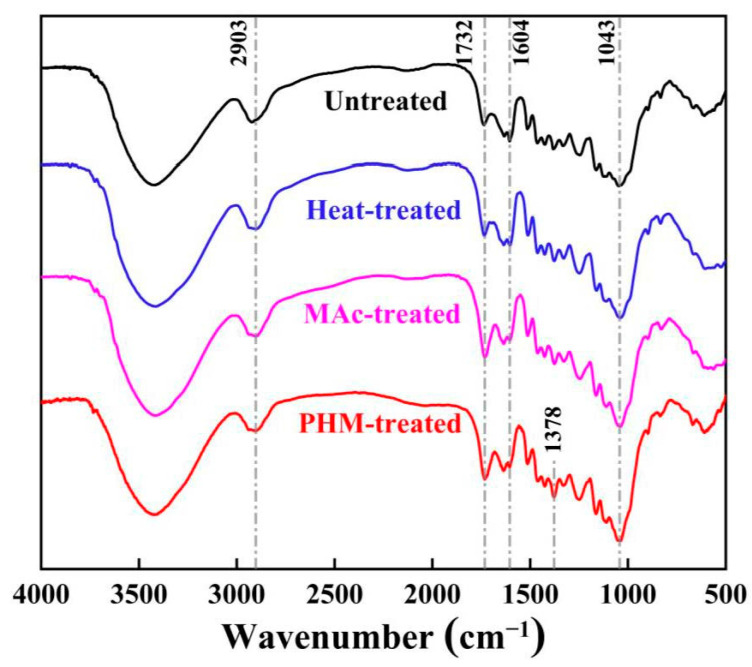
FT-IR spectra of untreated and treated (160 °C) bamboo powder samples.

The FT-IR spectra of bamboo powder untreated or treated in various ways were recorded in Figure 9. In the case of treated samples, the absorption of C=O stretching vibrations at 1604 cm^−1^ decreased due to the degradation of the xylan and glucomannan backbone during the thermal modification [51]. Meanwhile, the increase in C–H in-plane deformation of lignin at 1043 cm^−1^ was observed in the spectra of all treated materials. Bamboo has a higher relative lignin content with the thermal degradation of hemicellulose after heat treatment [52]. For MAc and PHM-treated samples, peaks at 2903 cm^−1^ and 1732 cm^−1^ associated with the methylene stretching and carbonyl stretching became more intense and discernible, indicating the occurred esterification between bamboo and MAc or PHM [53]. In addition, the enhanced absorption band at 1378 cm^−1^ related to C–CH_3_ of the HEMA units of PHM was observed in the spectrum of PHM-treated bamboo, representing the successful grafting of polymer chains onto the bamboo. Degradation of the hemicellulose and the increasing relative content of lignin on behalf of PHM modification’s effect were considered the main reason for the enhanced hydrophobicity. The result was in good agreement with the dimensional stability results mentioned above.

**Figure 10:**
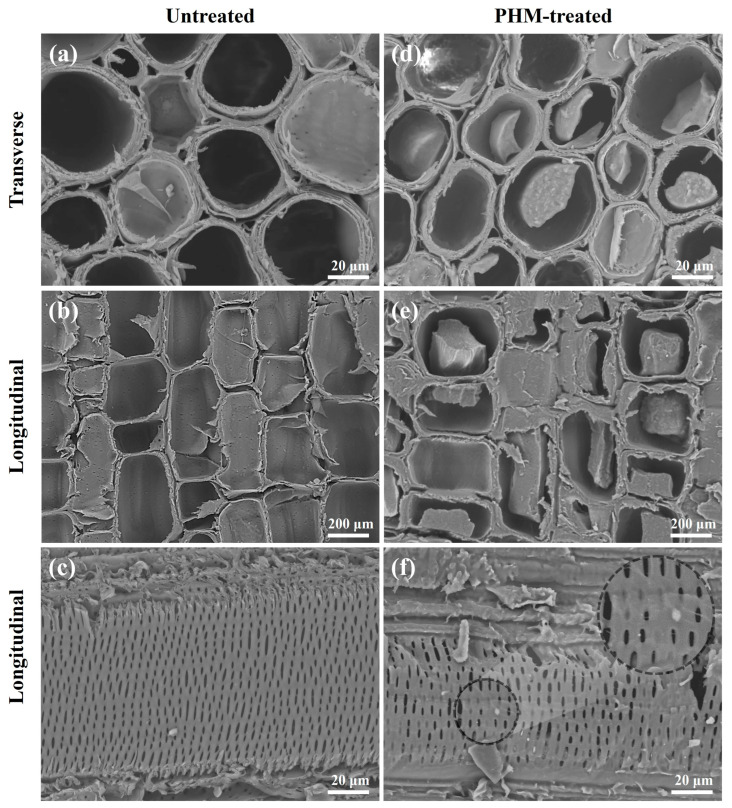
The morphology of PHM-treated LBL’s cross-section. Transverse section of untreated LBL (**a**) and PHM-treated LBL (**d**); Longitudinal section of untreated LBL (**b**,**c**) and PHM-treated LBL (**e**,**f**).

On the other hand, a hydrophobic polymer structure formed by the reaction of monomers physically filled in bamboo cells, hindering moisture migration inside the material. The cross-section of LBL was observed under a scanning electron microscope. Obviously, polymers formed and filled in modified LBL cells compared to the raw LBL (Figure 10). Monomers could enter the cell cavity of bamboo during the impregnation process and copolymerize to form PHM, which blocks water migration inside the material. This reaction behavior might be the main reason for the improved dimensional stability.

Dense pits were found on the cell wall when observing the longitudinal section of untreated LBL (Figure 10c). These pits provided channels for small molecule agents to enter the adjacent cells. Besides, the existence of PHM could be observed from the longitudinal section of the modified material, polymers formed and blocked the pits (Figure 10f). The modifier could enter the cell lumen, diffuse to the surrounding cells through the pits, and complete the polymerization in both the cell cavities and cell walls to achieve the filling effect.

## 4. Conclusions

This study established a dual modification method that uses a hydrophobic polymer with low deformation to physically fill bamboo cells with the elimination of hydroxyl groups via esterification to improve the dimensional stability of LBL. A crosslinked copolymer PHM was successfully synthesized in an aqueous solution. FT-IR and ^13^C-NMR identified their morphology and chemical structure. DSC analysis and swelling tests revealed that elevated temperatures facilitate PHM’s crosslinking, which results in ideal dimensional stability. Using PHM on LBL improved its hydrophobicity; anti-swelling properties decreased hydrophilic substances; and filled bamboo cells contributed to this sufficient improvement. The ASE of the modified LBL was up to 46.0%. PHM modification of LBL provided a novel approach to effectively improving its hydrophobicity and dimensional stability. LBL’s anti-mold performance and the efficient utilization of MAh will be researched in forthcoming studies.

## Figures and Tables

**Table 1:** Effect of reaction temperature on the physical properties of polymers.

Sample	Temperature (°C)	VG (%/%)	ML (%)
PHM-100	100	1.70 ± 0.34	0.85 ± 0.64
PHM-120	120	1.01 ± 0.31	6.65 ± 0.72
PHM-140	140	1.66 ± 0.68	4.46 ± 1.08
PHM-160	160	1.09 ± 0.47	1.85 ± 0.30
PHM-180	180	0.75 ± 0.67	5.50 ± 1.32

## Data Availability

The data presented in this study are available on request from the corresponding author.

## Supplementary Materials

The following supporting information can be downloaded at: https://www.mdpi.com/article/10.3390/polym15122637/s1, Figure S1: FT-IR spectra of MAh aqueous solution heated at different temperatures; Table S1: Surface free energy of untreated and treated bamboo surfaces; Table S2: Water contact angle hysteresis on bamboo surface; Table S3: The bending mechanical properties of LBL.

## References

[1] Liu S., Gao D., Xie Y., Chen B. (2022). Experimental Study and Theoretical Analysis of Side-Pressure Laminated Bamboo Lumber Columns under Axial Compression. Sustainability.

[2] Kelkar B.U., Sharma S.K., Shukla S.R. (2021). Comparative Performance of Phenol Formaldehyde-Bonded Laminated Bamboo Lumber and Bamboo Strand Lumber Prepared from Four Different Bamboo Species. J. Trop. For. Sci..

[3] Wang Z., Li H., Yang D., Xiong Z., Sayed U., Lorenzo R., Corbi I., Corbi O., Hong C. (2021). Bamboo node effect on the tensile properties of side press-laminated bamboo lumber. Wood Sci. Technol..

[4] Amada S., Ichikawa Y., Munekata T., Nagase Y., Shimizu H. (1997). Fiber texture and mechanical graded structure of bamboo. Compos. Part B Eng..

[5] Wang F.P., Li S., Wang L. (2017). Fabrication of artificial super-hydrophobic lotus-leaf-like bamboo surfaces through soft lithography. Colloids Surf. A-Physicochem. Eng. Asp..

[6] Guan M., Huang Z., Zhu D. (2022). The effect of ultrasonic process on the shear strength and the microstructure of the bonding interface of laminated bamboo lumber. Eur. J. Wood Wood Prod..

[7] Jin C.D., Li J.P., Han S.J., Wang J., Sun Q. (2014). A durable, superhydrophobic, superoleophobic and corrosion-resistant coating with rose-like ZnO nanoflowers on a bamboo surface. Appl. Surf. Sci..

[8] Wang J., Wang H., Wu X., Zhang Y., Jiang J., Han S., Sun F. (2021). Anti-mold activity and reaction mechanism of bamboo modified with laccase-mediated thymol. Ind. Crops Prod..

[9] Chen J., Ma Y., Lin H., Zheng Q., Zhang X., Yang W., Li R. (2019). Fabrication of Hydrophobic ZnO/PMHS Coatings on Bamboo Surfaces: The Synergistic Effect of ZnO and PMHS on Anti-Mildew Properties. Coatings.

[10] Gao X., Su L., Jiang G., Pang J., Lin L. (2020). Dimensional Stability of Lotus Leaf-like Nanostructure Superhydrophobic Bamboo by Modification Using Xylan. Bioresources.

[11] Rao J., Jiang J., Prosper N.K., Yang X., Liu T., Cai W., Wang H., Sun F. (2019). Combination of polyethylene glycol impregnation and paraffin heat treatment to protect round bamboo from cracking. R. Soc. Open Sci..

[12] Huang S., Jiang Q., Yu B., Nie Y., Ma Z., Ma L. (2019). Combined Chemical Modification of Bamboo Material Prepared Using Vinyl Acetate and Methyl Methacrylate: Dimensional Stability, Chemical Structure, and Dynamic Mechanical Properties. Polymers.

[13] Li W., Liu M., Zhai H., Wang H., Yu Y. (2020). Preparing highly durable bamboo materials via bulk furfurylation. Constr. Build. Mater..

[14] Zhang W., Zhou J., Cao Z., Wu X., Wang H., Han S., Zhang Y., Sun F., Zhang T. (2022). In Situ Construction of Thermotropic Shape Memory Polymer in Wood for Enhancing Its Dimensional Stability. Polymers.

[15] Wang Z.X., Han X.S., Wang S.J., Lv Y., Pu J. (2020). Enhancing the thermal stability, water repellency, and flame retardancy of wood treated with succinic anhydride and melamine-urea-formaldehyde resins. Holzforschung.

[16] Wang X.Z., Chen X.Z., Xie X.Q., Cai S., Yuan Z., Li Y. (2019). Multi-Scale Evaluation of the Effect of Phenol Formaldehyde Resin Impregnation on the Dimensional Stability and Mechanical Properties of Pinus Massoniana Lamb. Forests.

[17] Gallorini M., Cataldi A., Di Giacomo V. (2014). HEMA-induced cytotoxicity: Oxidative stress, genotoxicity and apoptosis. Int. Endod. J..

[18] Kwon S., Oh K., Shin S.J., Lee H.L. (2020). Effects of hydroxyethyl methacrylate comonomer in styrene/acrylate latex on coating structure and printability. Prog. Org. Coat..

[19] Liang X.-Y., Wang L., Wang Y.-M., Ding L.-S., Li B.-J., Zhang S. (2017). UV-Blocking Coating with Self-Healing Capacity. Macromol. Chem. Phys..

[20] Wu H., Yang X., Rao J., Zhang Y., Sun F. (2018). Improvement of Bamboo Properties via In Situ Construction of Polyhydroxyethyl Methylacrylate and Polymethyl Methylacrylate Networks. Bioresources.

[21] Huang Y., Li G., Chu F. (2019). In situ polymerization of 2-hydroxyethyl methacrylate (HEMA) and 3-(methacryloxy)propyltrimethoxysilane (MAPTES) in poplar cell wall to enhance its dimensional stability. Holzforschung.

[22] Redfearn H.N., Goddard J.M. (2022). Antioxidant and dissociation behavior of polypropylene-graft-maleic anhydride. J. Appl. Polym. Sci..

[23] Techawinyutham L., Frick A., Siengchin S. (2016). Polypropylene/Maleic Anhydride Grafted Polypropylene (MAgPP)/Coconut Fiber Composites. Adv. Mech. Eng..

[24] Essoua G.G.E., Blanchet P., Landry V., Beauregard R. (2015). Maleic Anhydride Treated Wood: Effects of Drying Time and Esterification Temperature on Properties. Bioresources.

[25] Teaca C.A., Bodirlau R., Spiridon I. (2014). Maleic anhydride treatment of softwood-effect on wood structure and properties. Cellul. Chem. Technol..

[26] He M.M., Xu D.D., Li C.G., Ma Y., Dai X., Pan X., Fan J., He Z., Gui S., Dong X. (2020). Cell Wall Bulking by Maleic Anhydride for Wood Durability Improvement. Forests.

[27] El Nazer H.A., Elgohary E.A., Rabie S.T. (2018). Photostability, thermal and mechanical properties of poly(vinyl chloride)/novel acrylate terpolymer blend. J. Vinyl Addit. Technol..

[28] Mahmoodzadeh F., Ghorbani M., Jannat B. (2019). Glutathione and pH-responsive chitosan-based nanogel as an efficient nanoplatform for controlled delivery of doxorubicin. J. Drug Deliv. Sci. Technol..

[29] Zhang W.P., Lu Y.H., Khanal S., Xu S.A. (2018). Effects of compatibilizers on selected properties of hdpe composites highly filled with bamboo flour. Wood Fiber Sci..

[30] Pavarelli G., Ochoa J.V., Caldarelli A., Puzzo F., Cavani F., Dubois J.-L. (2015). A New Process for Maleic Anhydride Synthesis from a Renewable Building Block: The Gas-Phase Oxidehydration of Bio-1-butanol. ChemSusChem.

[31] Pamfil D., Schick C., Vasile C. (2014). New Hydrogels Based on Substituted Anhydride Modified Collagen and 2-Hydroxyethyl Methacrylate. Synthesis and Characterization. Ind. Eng. Chem. Res..

[32] Shogren R.L. (2003). Rapid preparation of starch esters by high temperature/pressure reaction. Carbohydr. Polym..

[33] He W.-S., Ma Y., Pan X.-X., Li J.-J., Wang M.-G., Yang Y.-B., Jia C.-S., Zhang X.-M., Feng B. (2012). Efficient Solvent-Free Synthesis of Phytostanyl Esters in the Presence of Acid-Surfactant-Combined Catalyst. J. Agric. Food Chem..

[34] Liu Y.-w., Wang P., Wang J., Xu B., Xu J., Yuan J.-G., Yu Y.-Y., Wang Q. (2020). Transparent and tough poly(2-hydroxyethyl methacrylate) hydrogels prepared in water/IL mixtures. New J. Chem..

[35] Yan S., Gao X., Wang Z., Gao C. (2012). Antifouling and antibacterial improvement of surface-functionalized poly(vinylidene fluoride) membrane prepared via dihydroxyphenylalanine-initiated atom transfer radical graft polymerizations. J. Membr. Sci..

[36] Ptak S., Zarski A., Antczak T., Kapusniak J. (2016). Esterification of potato starch with oleic acid in the presence of lipase from Candida antarctica in a microwave field and under conventional heating. Polimery.

[37] Mahmoodian H., Moradi O., Shariatzadeha B., Salehf T.A., Tyagi I., Maity A., Asif M., Gupta V.K. (2015). Enhanced removal of methyl orange from aqueous solutions by poly HEMA–chitosan-MWCNT nano-composite. J. Mol. Liq..

[38] Nassar N., Whitehead F., Istivan T., Shanks R., Kasapis S. (2021). Manipulation of the Glass Transition Properties of a High-Solid System Made of Acrylic Acid-N,N′-Methylenebisacrylamide Copolymer Grafted on Hydroxypropyl Methyl Cellulose. Int. J. Mol. Sci..

[39] Muoz-García R.O., Hernández M.E., Ortiz G.G., Fernández V.V., Arellano M.R., Sánchez-Díaz J.C. (2019). A novel polyacrylamide-based hydrogel crosslinked with cellulose acetate and prepared by precipitation polymerization. Química Nova.

[40] Kariper I.A. (2017). The Release of Doxorubicin’s Active Ingredient from the Hydrogels with Poly (HEMA/Acrylamide/Itaconic acid) and Their Biological Function. Indian J. Pharm. Educ. Res..

[41] Gupta V.K., Tyagi I., Agarwal S., Sadegh H., Shahryari-ghoshekandi R., Yari M., Yousefi-nejat O. (2015). Experimental study of surfaces of hydrogel polymers HEMA, HEMA–EEMA–MA, and PVA as adsorbent for removal of azo dyes from liquid phase. J. Mol. Liq..

[42] Esselin N., Portolan F., Domloge N., Clark R.B., Musa O.M., Pilard J.-F. (2015). Offline Monitoring of Hydroxyethyl Methacrylate and 3-Dimethylaminopropyl Methacrylamide Copolymerization: Correlation between FTIR and GC Quantifications. Spectrosc. Lett..

[43] Dagys L., Klimkevičius V., Klimavicius V., Aidas K., Makuška R., Balevicius V. (2020). CP MAS kinetics in soft matter: Spin diffusion, local disorder and thermal equilibration in poly(2-hydroxyethyl methacrylate). Solid State Nucl. Magn. Reson..

[44] Karakus G., Zengin H.B., Polat Z.A., Yenidunya A.F., Aydin S. (2013). Cytotoxicity of three maleic anhydride copolymers and common solvents used for polymer solvation. Polym. Bull..

[45] Fu H., Yan C., Wei X. (2013). Study on Chlorination of Maleic Anhydride Grafted Polypropylene. Polym. Polym. Compos..

[46] Prashantha K., Rashmi B.J., Lee J.H. (2013). Preparation and characterization of carbon nanotube filled poly (2-hydroxyethylmethacrylate) nanocomposites. High Perform. Polym..

[47] Huang Y.Q., Yu Y., Zhang C., Wang X., Yang Z., Yang Y. (2022). Investigation of the relationship between surface colour, contact angle and chemical properties of heat-treated bamboo. Wood Mater. Sci. Eng..

[48] Li T., Cheng D.L., Walinder M.E.P., Zhou D.-G. (2015). Wettability of oil heat-treated bamboo and bonding strength of laminated bamboo board. Ind. Crops Prod..

[49] Lee S.H., Wang S. (2006). Biodegradable polymers/bamboo fiber biocomposite with bio-based coupling agent. Compos. Part A Appl. Sci. Manuf..

[50] Geng Y., Pei X., He X., Li P., Wu Y., Zuo Y. (2018). Preparation and Characterization of Esterified Bamboo Flour by an In Situ Solid Phase Method. Polymers.

[51] Guo X., Wu Y., Yan N. (2018). In situ micro-FTIR observation of molecular association of adsorbed water with heat-treated wood. Wood Sci. Technol..

[52] Wang Y., Huang Y., Xue J., Peng Y., Cao J. (2021). Effects of heat treatment temperatures on the photostability of Moso bamboo during accelerated UV weathering. Wood Mater. Sci. Eng..

[53] Mokhena T.C., John M.J. (2020). Esterified cellulose nanofibres from saw dust using vegetable oil. Int. J. Biol. Macromol..

