# Supplementary material for: Exploration of Optimal Reaction Conditions for Constructing Hydrophobic Polymers with Low Deformation to Facilitate the Dimensional Stability of Laminated Bamboo Lumber
Source: Polymers (Basel). 2023 Jun 9;15(12):2637. doi: 10.3390/polym15122637 (PMC10302235; doi:10.3390/polym15122637)
Supplement: Supplementary file 1 [file polymers-15-02637-s001.zip › polymers-2345656-supplementary.pdf]

Supplementary Materials

Exploration of Optimal Reaction Conditions for Constructing Hydrophobic Polymers with Low Deformation to Facilitate the Dimensional Stability of Laminated Bamboo Lumber

Jianchao Zhou [1], Li Jin [1], Xinxing Wu [1,*], Hui Wang [1], Shuaibo Han [1], Yan Zhang [1] and Fangli Sun [1,*]

[1] College of Chemistry and Materials Engineering, National Engineering & Technology Research Center for the Comprehensive Utilization of Wood-Based Resources, Zhejiang A&F University, Hangzhou 311300, China

* Correspondence: Correspondence: xinxingwu@zafu.edu.cn (X.W.); sunfangli@163.com (F.S.)

Surface free energy analysis

Based on the contact angles (t=3s) for deionized water and diiodomethane, Surface free energy of bamboo was determined according to the Owens-Wendt theory. Equations are as follow:

$$\gamma_S = \gamma_S^P + \gamma_S^D \tag{S1}$$

$$\gamma_L(1 + cos\theta) = 2(\sqrt{\gamma_S^D \gamma_L^D} + \sqrt{\gamma_S^P \gamma_L^P}) \tag{S2}$$

Where γ_S and γ_L represent the surface free energy (surface tension) of bamboo and probing liquid, respectively. The superscripts D and P denote dispersion components and polar components of surface energy. θ is the contact angle between bamboo surface and probing liquid.

Table S1. Surface free energy of untreated and treated bamboo surfaces.

	**Deionized water**	**Diiodomethane**	**Untreated bamboo**	**Heat-treated bamboo (140 °C)**	**PHM-treated bamboo (140 °C)**
**Dispersion components (mJ/m^2)**	21.8	48.5	27.9	24.8	5.9
**Polar components (mJ/m^2)**	51.0	2.3	18.5	12.2	1.8
**Surface tension (mJ/m^2)**	72.8	50.8	46.4	37.0	7.7

Dynamic contact angle values

Table S2. Water contact angle hysteresis on bamboo surface.

	**Untreated bamboo**	**Heat-treated bamboo (140 °C)**	**PHM-treated bamboo (140 °C)**
**Advancing contact angle (°)**	63.8	117.4	121.3
**Receding contact angle (°)**	34.4	89.9	106.9
**Contact angle hysteresis (°)**	29.4	27.5	14.4

Mechanical behavior of LBL

Table S3. The bending mechanical properties of LBL.

	**Bending strength (MPa)**	**Elastic modulus (MPa)**
**Raw LBL**	120.5	6901.2
**PHM-modified LBL**	117.3	6692.8

FT-IR Analysis

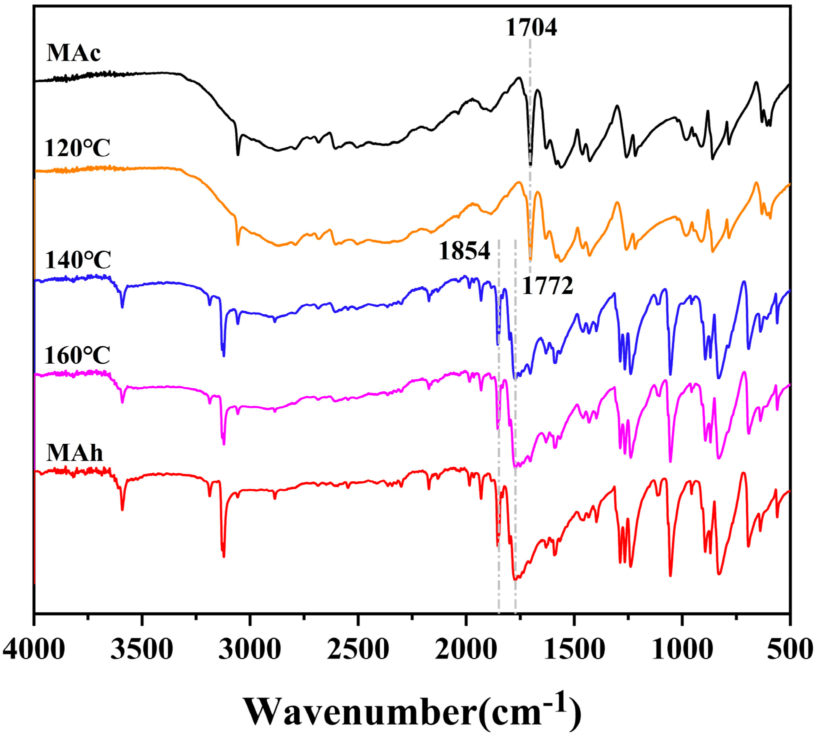

Figure S1. FT-IR spectra of MAh aqueous solution heated at different temperatures.
